# Preoperative prone position exercises: a simple and novel method to improve tolerance to kyphoplasty for treatment of single level osteoporotic vertebral compression fractures

**DOI:** 10.1186/s12891-017-1843-3

**Published:** 2017-11-21

**Authors:** Guangzhou Li, Hao Liu, Qing Wang, Dejun Zhong

**Affiliations:** 1 Department of Spine Surgery, the Affiliated Hospital of South-west Medical University, No.25 Taiping St, Luzhou, Sichuan 646000 China; 2 Department of orthopedics, Sichuan University West China Hospital, Sichuan Province, Chengdu, China

**Keywords:** Kyphoplasty, Prone position, Exercises, Osteoporotic vertebral compression fractures

## Abstract

**Background:**

The proper choice of anesthesia for kyphoplasty remains controversy. There are only a few clinical studies specially focusing on and giving detailed information about this treatment under local anesthesia with or without conscious sedation. To evaluate the effect of preoperative prone position exercises on patient tolerance to percutaneous kyphoplasty under local anesthesia.

**Methods:**

Eighty-three patients with single level osteoporotic vertebral compression fractures were nonrandomly assigned to undergo percutaneous kyphoplasty under local anesthesia with preoperative prone position exercises or without. The number of procedure with or without a pause, need for intravenous sedation, and patient satisfactory were recorded and analyzed. Clinical outcomes were assessed using the visual analog scale and the Oswestry Disability Index. The follow-up time was 6 months.

**Results:**

The baseline characteristics of both groups were comparable. The number of procedure without a pause in the exercises group was more than the control group (30/42 patients and 10/41 patients, respectively, *P* < 0.001), and fewer patients required intravenous sedation in the exercises group (7/42 and 28/41, respectively, *P* < 0.001). Patients in the exercises group were more satisfied compared to the control group (41/42 and 32/41, respectively, *P* < 0.01). There were no significant differences between the two groups with regard to improvement in pain and functional scores at all postoperative intervals.

**Conclusions:**

Prone position exercises may improve patient tolerance and satisfaction and reduce the need for intravenous sedation for those with single level vertebral compression fracture undergoing kyphoplasty under local anesthesia. We expect large sample size and multi-center randomized controlled trial studies to be conducted.

## Background

Kyphoplasty (KP), developed from vertebroplasty (VP), is a minimally invasive procedure for the treatment of several vertebal diseases [1–3]. Osteoporotic vertebral compression fractures (OVCF) is the mostly common pathologic condition treated. Compared with VP, KP has a better effect on reducing kyphosis and restoring the height of the vertebral body [4, 5]. However, KP is technically more demanding and complex, and patients undergoing such procedure are generally older and may have numerous illnesses simultaneously, which may especially impair the ability to lie in a prone position for a long time during this procedure, resulting in abandoning the surgery and increasing the risk of anesthesia-related complications [6, 7]. Many authors performed this treatment with patients under general anesthesia [8, 9]. The proper choice of anesthesia for kyphoplasty remains controversy. On the other hand, a few clinical studies reported KP under local anesthesia without detailed information [4, 10].

Ideally, KP should be performed with the patient under local anesthesia with or without conscious sedation. This study was undertaken to evaluate the effect of preoperative prone position exercises (PPE) on patient tolerance to KP under local anesthesia and to determine if such exercises would improve postoperative clinical outcome.

## Methods

### Study design

Between February 2010 and December 2012, 83 patients with single level painful OVCF not more than 4 weeks old were enrolled in the study. Patients with previous surgery of the spine, psychological diseases, or neurologic deficits, were excluded from participation. This study was approved by Affiliated Hospital of South-west Medical University review board. In our department, two senior spinal surgeons (Q.W. and D.Z.) performed KP in two treatment groups, respectively. One surgeon (Q.W.) preferred that all patients should do PPE, whereas the other one (D.Z.) did not recommend it to patients. So, patients were assigned to undergo KP with PPE or without depending on the surgeon they were referred. Consequently, 42 patients were included in the PPE group (exercises group), and 41 patients in the without PPE group (control group).

A comprehensive assessment of the patients’ general condition was performed after admission. On the day before operation, the purpose and procedures involved in the study were fully explained to the patients, and informed consent was obtained before data collection. The PPE was essentially a tolerance test. In the exercises group, with the participators lying in the prone position, one or two soft pillow was placed under their chest. The patients were placed in prone position for 40 to 60 min, depending on tolerance of oneself, and an investigator recorded the duration and if there was any discomfort. Patients did prone exercises twice. If someone who could not maintain the prone position for 40 min, we placed him/her prone for 20–30 min. In the control group, patients did not do PPE, and no any other exercises given.

### Surgical procedures

Surgical procedure was performed under local anesthesia with the patient positioned prone on a radiolucent table with his/her spine extended by chest and pelvic bolsters. C-arm fluoroscopy was used throughout the procedure.

In addition to local anesthesia using 10 ml of 1% lidocaine, if the patient complained inadequate anesthesia during surgical procedure, he or she would be sedated using intravenous fentanyl 0.5 μg/kg and intravenous propofol 0.5 mg.kg^−1^, and the depth of sedation was monitored by anesthetists to make sure the patient in a safe condition.

With fluoroscopy visualization, transpedicular (in the lumbar vertebrae) or extrapedicular (in the thoracic vertebrae) puncture was performed unilaterally. After reaching the posterior margin of the vertebral body, the bone needle was exchanged for a working cannula. A Balloon with radio-opaque media was inserted into the fractured vertebral body to restore the damaged vertebral body until adequate height restoration and kyphosis correction were obtained or when the inflation pressure reached 220 psi. Then, the balloon was deflated and withdrawn, and the resultant intravertebral cavity was filled with polymethylmethacrylate (PMMA) cement. Monitoring was continued on the ward for 8 h after the operation.

### Outcome assessment

Clinical data were prospectively collected and analyzed by one independent observer. On the first postoperative day (the day after the operation), patients were asked whether they were satisfied with such treatment and whether they would undergo the procedure again. The number of interventions that had to be discontinued and the patients who received intravenous sedation were recorded and analyzed.

Pain was evaluated using a visual analogue scale (VAS: 0 = no pain at all, 10 = worst pain imaginable) before and after operation (the first postoperative day), continuing postoperatively at 3 and 6 months. The functional outcome was assessed using the Oswestry Disability Index (ODI) preoperatively and postoperatively at 3 and 6 months.

### Statistical analysis

For quantitative variables (the VAS and ODI scores), the groups were compared preoperatively and postoperatively using a paired student’s t-test, and the comparisons between the two groups were made by student’s t-test. For qualitative variables, the comparisons were made by Chi-square or Fischer’s exact test where appropriate. Analyses were performed using SPSS statistical software (Version 13.0, SPSS Inc., Chicago, IL). For all comparisons, a *P* value < 0.05 was considered significant.

## Results

The baseline characteristics of both groups were comparable (Table 1). After 6 months, no patient was lost to follow-up and was excluded from the study. Before operation, one patient with severe thoracic kyphotic deformity just did PPE for 25 min each time, and the others 41 tolerated PPE well without discomfort.

**Table 1:** Characteristics of the study population

	Exercises group	Control group
No.patients	42	41
Patient age(y)(range)	71.4 ± 8.5(56–92)	70.51 ± 7.8(56–84)
Sex[No.(%)]		
Male	11 (26.2)	13 (31.7)
Female	31 (73.8)	28 (68.3)
Distribution of the fractured vertebrae [No.(%)]		
Thoracic(T6-T10)	8 (19.0)	9 (22.0)
Thoracolumbar(T11-L2)	30 (71.4)	27 (65.9)
Lumbar(L3-L5)	4 (9.5)	5 (12.2)
Chronic obstructive pulmonary disease	13	11
Coronary heart disease	8	9
Diabetes	7	7
Hypertension	17	16
Thoracic kyphosis deformity	11	12

All operations were performed with patients under local anesthesia with or without conscious sedation. No complications related to procedures, anesthesia and sedation occurred, which was confirmed by anesthetists’ recorded. No statistically significant difference was observed when the mean duration of operations was compared between the groups (43.8 ± 12.2 min for the exercises group and 47.3 ± 13.7 min for the control group, respectively).

The number of procedure without a pause in the exercises group was more than the control group (30/42 patients and 10/41 patients, respectively, *P* < 0.001), and fewer patients required intravenous sedation in the exercises group (7/42 and 28/41, respectively, *P* < 0.001). More patients in the exercises group were more satisfied compared to the control group (*P* < 0.01, Table 2).

**Table 2:** Comparison of procedure-related data and patient satisfaction using Chi- square or Fischer’s exact test

	Exercises group (*n* = 42)	Control group (*n* = 41)	*P* value
Procedure without a pause (yes/no)	30/12	10/31	*P* < 0.001
Need for intravenous sedation(yes/no)	7/35	28/13	*P* < 0.001
Patient willing to repeat(yes/no)	41/1	32/9	*P* < 0.01

The score of VAS decreased significantly in both groups (Table 3). In the exercises group, the mean pain score decreased from 7.8 ± 1.1 (range, 5–9) before operation to 2.4 ± 1.0 (range, 1–4) on the first postoperative day (*P* < 0.001) and to 2.3 ± 0.7 (range, 1–3) at 6 months after operation(*P* < 0.001); In the control group, VAS score improved from 7.6 ± 1.0 (range, 6–9) preoperatively to 2.4 ± 1.0 (range, 1–4) on the first postoperative day (*P* < 0.001) and to 2.4 ± 0.8 (range, 1–4) at 6 months postoperatively (*P* < 0.001). The ODI score had a similar trend. The mean ODI score decreased from 60.5 ± 6.4 (range, 48–72) preoperatively to 30.6 ± 4.9 (range, 22–42) (*P* < 0.001) and 30.0 ± 4.6 (range, 22–42) (*P* < 0.001) at 3 and 6 months, respectively, in the exercises group; The ODI score improved from 61.1 ± 5.4 (range, 48–72) preoperatively to 29.9 ± 4.1 (range, 24–41) (*P* < 0.001) and 30.1 ± 4.5 (range, 24–42) (*P* < 0.001) at 3 and 6 months, respectively, in the control group. There were no significant differences between the two groups with regard to improvement in pain and functional scores at all postoperative intervals.

**Table 3:** Comparison of preoperative and postoperative mean VAS scores

	Preoperation	The first postoperative day	3 months after operation	6 months after operation
Exercises group	7.8 ± 1.1	2.4 ± 1.0*	2.4 ± 0.9*	2.3 ± 0.7*
Control group	7.6 ± 1.0	2.4 ± 1.0*	2.5 ± 1.0*	2.4 ± 0.8*

Abbreviations: *VAS* visual analogue scale (VAS: 0 = no pain at all, 10 = worst pain imaginable)

## Discussion

As the ageing of the population of all over the world, the incidence of OVCF is likely to increase rapidly and provides a great challenge to health care workers and patients [11, 12]. KP has been proven to be an effective minimally invasive procedure for the treatment of OVCF [2–5]. However, KP usually is performed with the patient under general anesthesia [8, 9, 13–15]. This do cause a therapeutic dilemma that patients undergoing such procedure are generally older and may have numerous illnesses which may pose a higher risk for general anesthesia than the minimally invasive procedure per se [6, 7]. Ideally, KP is performed with the patient under local anesthesia.There are only a few clinical studies specially focusing on and giving detailed information about KP under local anesthesia with or without conscious sedation [4, 7, 10, 16, 17]. To the best of our knowledge, there is no report about preparing patients with PPE to study the effect on patient tolerance under local anesthesia and the clinical outcome in the literature so far.

The present study demonstrates that through PPE patients tolerated operation better, with fewer requiring intravenous sedation, and patients were more satisfied with such procedure. The reason why PPE improves patient tolerance under local anesthesia might be: In addition to poor physical function caused by the vertebral fracture and numerous illnesses simultaneously, psychosocial factors, including lack of confidence, low mood, anxiety, distress, may also play a key role in impairing the ability of patients with OVCF to lie in a prone position for a long time. Through the process of PPE, we built up patients’ physical adaptation to prone position and patients’ confidence, and thus improved patients’ psychological and physical tolerance to KP. During PPE, a kind of comprehensive intervention measures was implemented for preoperative preparation, including physical training, psychological support, and patient education. This reminds us that a combination of preoperative management, including physiotherapy, psychological support and patient education should be taken for such patients before operation.

Although the causes of pain in OVCF are multi-factorial, it is known that this is due mainly to micro-movement of the vertebral fracture [18]. Effective and rapid relief from pain and satisfactory clinical outcomes can be obtained after elimination of the microfractures, vertebral stabilization, and the chemical neurolytic and thermal neurolytic effect of the PMMA [5, 19]. All these could explain that although PPE improved patient tolerance and satisfaction, there were no statistically significant differences in pain alleviation and functional improvement between the two groups postoperatively. Due to various reasons, some authors still supported assisted sedation for KP or VP [7, 17, 20]. However, considering that patients with OVCF are generally older and may have numerous illnesses, reducing the use of additional sedation or general anesthesia means more safety and less potential risks for patients undergoing KP or VP treatment. Although our PPE did not improvement patients’ pain and functional scores compared with patients in the control group, it still might provide potential value to health care workers and patients.

PPE brings some advantages as follows: Firstly, it spares the use of additional intravenous sedation or general anesthesia with its potential complications [21, 22]. Secondly, it may help patients resume their daily activities early after operation. Thirdly, although we did not compare the cost of between the two groups, exercises group appears to be more cost-effective since potential adverse events related to intravenous sedation would be reduced.

Our study has a few limitations that should be mentioned. First, all patients in our study were with single level OVCF. For 2 or more than 2 levels OVCFs, the duration time of KP would be too long to use local anesthesia without intravenous sedation, and sometimes we initially recommended general anesthesia for them. So we did not enroll patients with 2 or more OVCF in the current study. Second, we did not randomize the patients between the groups because such randomization might cause ethical concerns. In our study, patients were allocated to different groups depending on the surgeons they were referred. So, any significant selection bias should have been fairly well mitigated. Third, the follow-up was 6 months. With no statistically significant differences in pain alleviation and functional improvement between the two groups during the 6-month follow-up, we did not expect that a long-term follow-up would bring significant differences. In addition, additional morbidities and mortality related to this elderly population may prohibit long-term follow-up. Other limitations of our study include the small sample size and non-multicenter study.

## Conclusions

Considering the limitation of the current study by its small number of patients and nonrandomized comparative design, we carefully suggest that PPE may improve patient tolerance and satisfaction and reduce the need for intravenous sedation for those with single level vertebral compression fracture undergoing KP under local anesthesia. However, to confirm and validate the results of our study, large sample size and multi-center randomized controlled trial studies should be conducted.

## Abbreviations

KP: Kyphoplasty
ODI: Oswestry Disability Index
OVCF: Osteoporotic vertebral compression fractures
PMMA: Polymethylmethacrylate
PPE: Prone position exercises
VAS: Visual analogue scale
VP: Vertebroplasty
